# Mechanisms of *Cryptococcus neoformans*-Mediated Host Damage

**DOI:** 10.3389/fimmu.2018.00855

**Published:** 2018-04-30

**Authors:** Arturo Casadevall, Carolina Coelho, Alexandre Alanio

**Affiliations:** ^1^Department of Molecular Microbiology and Immunology, Johns Hopkins School of Public Health, Baltimore, MD, United States; ^2^Institut Pasteur, Molecular Mycology Unit, CNRS UMR2000, Paris, France; ^3^Laboratoire de Parasitologie-Mycologie, Hôpital Saint-Louis, Groupe Hospitalier Lariboisière, Saint-Louis, Fernand Widal, Assistance Publique-Hôpitaux de Paris (AP-HP), Université Paris Diderot, Sorbonne Paris Cité, Paris, France

**Keywords:** *Cryptococcus*, cryptococcosis, disease, damage, macrophage, cytotoxicity

## Abstract

*Cryptococcus neoformans* is not usually considered a cytotoxic fungal pathogen but there is considerable evidence that this microbe can damage host cells and tissues. In this essay, we review the evidence that *C. neoformans* damages host cells and note that the mechanisms involved are diverse. We consider *C. neoformans*-mediated host damage at the molecular, cellular, tissue, and organism level. Direct mechanisms of cytotoxicity include lytic exocytosis, organelle dysfunction, phagolysosomal membrane damage, and cytoskeletal alterations. Cytotoxicity contributes to pathogenesis by interfering with immune effector cell function and disrupting endothelial barriers thus allowing dissemination. When *C. neoformans*-mediated and immune-mediated host damage is sufficient to affect homeostasis, cryptococcosis occurs at the organism level.

Anyone with expertise in routine laboratory tissue culture knows that fungal contamination rapidly kills mammalian cells *in vitro*. However, *Cryptococcus neoformans* is unusual among fungi in that it has minimal toxicity for animal cells in tissue culture, such that it is possible to maintain yeast cells and macrophages for days without major cytotoxicity for the latter. macrophage-like cells that have phagocytosed *C. neoformans* are capable of replicating and divide their yeast cargo among daughter cells (1). This implies that *C. neoformans* does not release major cytotoxic products, at least *in vitro*. Consistent with this notion, cryptococcal infections are not associated with tissue necrosis as seen in infections caused by other fungal pathogens, such as those caused by *Aspergillus* or Mucorales spp. In fact, cryptococcosis often shows many features of a chronic infection, and host death is often due to the effects of physical compression of tissue, and defects in resorption of cerebrospinal fluid (CSF) (possibly due to increased viscosity from fungal polysaccharide shedding into CSF) and overwhelming brain edema (2). While these observations might lead to the conclusion that *C. neoformans* infections are associated with minimal host damage, a review of available knowledge reveals otherwise. In this essay, we survey the available evidence that *C. neoformans* is capable of inflicting direct damage on host cells and tissues. We note that host damage following cryptococcal infection can come from microbe and the host (3, 4), with the latter culminating in a dramatic pathology known as Immune Reconstitution Inflammatory Syndrome (IRIS).

We consider damage at four levels of scale: molecular, cellular, tissue, and organism level. Molecular damage is that caused by enzymes or molecules produced by *C. neoformans*, which induces modifications of host molecules and cells and manifests itself at the molecular or organelle level. Cellular damages are those causing modifications of the architecture and structure of the host cells due to the toxic action of *C. neoformans*. Tissue damages cause anatomical and functional disorganization beyond cellular injury. Together, these combine to produce the disease of cryptococcosis at the organism level. We recognize that these are not independent, since molecular damage leads to cellular damage, cellular damage leads to tissue damage, and all three combine to produce organismal damage. Furthermore, we note that a process of *C. neoformans* can damage the host at more than one level. For example, cryptococcal phospholipase can cause molecular damage by destroying surfactant molecules (5) while also being a potential cause of cellular damage at the level of macrophage (6). Despite these important caveats, these mechanisms are sufficiently distinct that it is possible to discuss them separately. Our goal is integrating them to produce a holistic view of *C. neoformans*-mediated host damage into a new synthesis for approaching cryptococcal pathogenesis (Figure 1).

**Figure 1:**
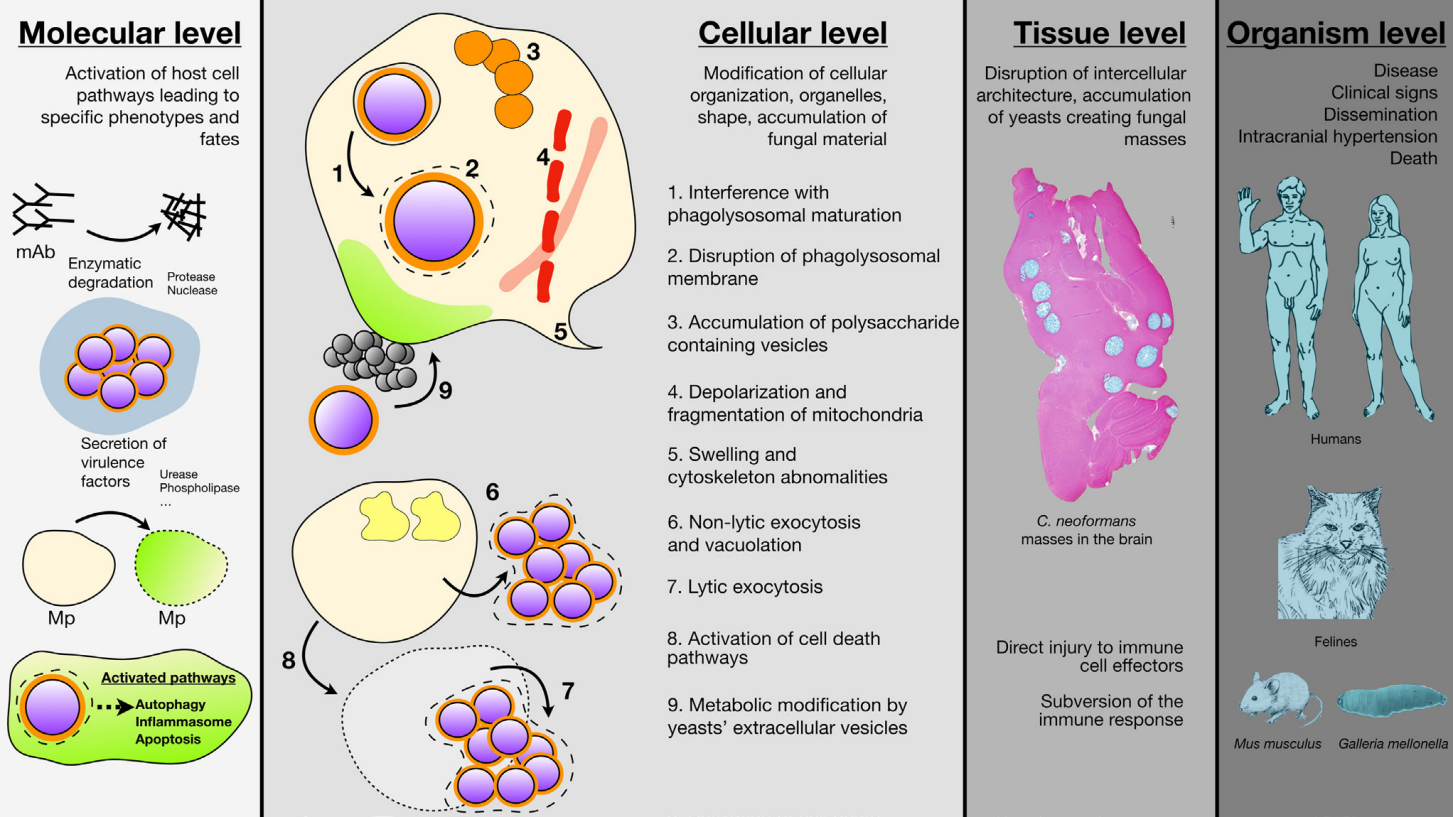
Schematic representation of the different *Cryptococcus neoformans*-mediated cell host damages are various scales. Damage at the molecular level results from the secretion of various enzyme by *C. neoformans* (proteases, nuclease, urease, phospholipase) that degrade host molecules such as antibodies and/or modify cells membranes. *C. neoformans* ingestion is also able to trigger autophagy, apoptosis, and cell death in the host (mAb, monoclonal antibodies; Mp, macrophages). Damage at the cellular level involves modification of cellular compartments such as accumulation of polysaccharide vacuoles (1), inhibition of phagolysosomal maturation (2), phagolysosomal leakage (3), mitochondrial fission and depolarization (4), swelling and cytoskeleton abonomalities (5) or metabolic modification due to *C. neoformans* vesicles secretions (6), *C. neoformans* engulfment resulted also in non-lytic (7), or lytic (8) exocytosis. Damage at the tissue level consisted typical cryptococcal lesions in the brain parenchyma after intravenous inoculation of *C. neoformans* to outbred mice (sacrifice seven days after inoculation). No granuloma and accumulation of yeast masses without inflammatory cells can be observed engendering tissue disorganization. Coloration Alcian Blue (magnification 4×). Damage at the organism level combines to produce the clinical signs associated with cryptococcal diseases in humans with dissemination and neurological abnormalities as the most severe clinical presentation leading to death. Felines are also naturally susceptible to cryptococcosis with localized to disseminate infections. *Mus musculus* and *Galleria mellonella* are well established organisms for experimental models of infection that help understanding the pathophysiology of the disease and the biology of the yeast in relation to the host.

## Molecular Damage

In the section for molecular damage, we consider how cryptococcal products damage host molecules (Figure 1). *C. neoformans*-mediated molecular damage enhances its likelihood of survival in tissues. As a soil-dwelling organism that obtains its nutrition from digesting material in the environment, *C. neoformans* secretes a large suite of enzymes with the potential to degrade host molecules (7). Among all enzymes produced by the fungus, the major candidates as mediators of host toxicity at the molecular level are proteases, urease, phospholipase, and nuclease (7). *C. neoformans* can metabolize immunoglobulins and complement proteins for growth as these compounds are presumably degraded by released proteases (8). Hence, proteases may interfere with host defense mechanisms by cleaving immunologically important molecules and directly damaging effector cells. Cryptococcal serine proteases promote increased blood–brain barrier (BBB) permeability (9), which may help in the process of brain infection. Although not directly related to host damage *C. neoformans* releases a protease that cleaves a peptide, which functions as quorum-sensing molecule to increase virulence (10). Urease is a virulence factor for *C. neoformans* (11), which is important for brain invasion (12). The mechanism by which urease promotes brain invasion could involve catalyzing the hydrolysis of urea to ammonia to locally damage endothelial cells in the brain vasculature. Another group of enzymes involved in the pathogenesis of *C. neoformans* are phospholipases. *C. neoformans* produces both phospholipase B and C (6, 13–17). Phospholipases cleave phospholipids, which in turn allow them to damage membranes. Phospholipase-deficient *C. neoformans* manifest delayed intracellular replication, which could result in better maintenance of phagosomal membrane integrity and subsequent enhanced fungal control (6). *In vitro* phospholipase-mediated cleaves surfactant and promotes the attachment of *C. neoformans* to human lung epithelial cells, a process *in vivo* could promote pulmonary infection (5). Ingestion of *C. neoformans* results in the activation of autophagy initiation complex pathways, which results in a global reprogramming of host kinase signaling (18).

## Cellular Damage

By cellular damage, we consider mechanisms for cytotoxicity. *C. neoformans*-mediated cytotoxicity contributes to establishment of disease *via* at least two major mechanisms. First, damage to host immune system in tissue, which inflicts damage to immune system, the surrounding tissues and may cause symptoms to the host while ultimately allowing persistence of infection. Second, damage to the endothelial cells in the brain vasculature (possibly in other organs as well), precedes invasion of the central nervous system to cause meningoencephalitis, the most common life-threatening form of cryptococcosis. Interaction of *C. neoformans* with the epithelial barriers is transient, and internalization of *C. neoformans* by epithelial cells is rarely observed. The airway epithelium is critical to trigger initial inflammatory response to the inhaled spores or yeast (19) and can produce surfactant, which agglutinates yeast cells (20). Potentially important to the pathogenesis of disease are interactions of *C. neoformans* with neurons or (micro)glial cells and their potential to cause neurological dysfunction but so far this remains an enigma (21). Therefore, we focus our discussion on cytotoxic damage primarily on phagocytic cells and endothelial cells and describe several forms of damage that can be inflicted on host cells (Figure 1).

1. *Interference with phagolysosomal maturation*: after ingestion of *C. neoformans* by macrophage, yeasts resides in an acidic phagosome that has maturation markers such as Lamp1 (22). Initially, *C. neoformans* was thought to not interfere with phagosome maturation similar to other intracellular pathogens such as *Mycobacterium tuberculosis*. However, recent studies indicate that ingestion of live but not dead *C. neoformans* cells is associated with the early removal of phagosome maturation markers Rab5 and Rab11 and interference with proteolytic activity and calcium fluxes (23). Interference with phagolysosome maturation would have the effect of interfering with its microbicidal activity, which in turn would promote fungal intracellular replication.
2. *Phagolysosomal leakage*: electron microscopic studies revealed damaged membranes in phagosomes containing *C. neoformans* (24). Phagolysosomal leakage was confirmed with fluores-cence labeled microscopy (25). Leakage of phagosome components into the cytoplasm interferes with microbicidal activity of this organelle but may also trigger the inflammasome (26). The amount of phagosomal leakage is modulated by IFN gamma (27), and likely other cytokines, with consequences to the amount of host cell damage and death.
3. *Accumulation of polysaccharide-containing vesicles in macrophage: C. neoformans* infection in macrophage is accompanied by the accumulation of vesicles, which contain fungal polysaccharide (24). The presence of large numbers of vesicles in the macrophage cytoplasm can give the impression of “holes” and these cells were named “hueco cells” after the Spanish term for hole (24). These intracytoplasmic vesicles appear to bud from the cryptococcal phagolysosome (24). Whether vesicle accumulation causes direct damage to the host cell is not known but their presence crowds out the cytoplasm and could impair cellular function. In addition, the generation of such large number of vesicles must tax lipid reserves (as composed of lipid bilayer), which could put a stress on membrane generation and repair.
4. *Interference with organelle function*: upon detection of intruder pathogenic microbes, macrophage undergo a series of concerted metabolic changes ranging from shift to a glycolytic metabolism to adaptation of the rate of protein synthesis or autophagy. Cellular quality control is a method to detect pathogen interference with organelle and cellular function and, therefore, is a mechanism of immune surveillance. When *C. neoformans* infects murine macrophage mitochondria potential is decreased and protein synthesis is impaired (28, 29). The presence of extracellular yeast could also lead to chromosomal aberrations and cell cycle impairment in murine macrophage (28).
5. *Host cell swelling and cytoskeleton alterations*: several decades ago, cryptococcal polysaccharide was reported to produce direct swelling effects on host cells (30–32). Although the biochemical pathway is still unknown, there is evidence that *C. neoformans* can induce changes in the cellular cytoskeleton. Human brain microvascular endothelial cells exposed to *C. neoformans* manifested changes in membrane ruffling, morphological changes in the nucleus, and swelling of the mitochondria and endoplasmic reticulum (33). These changes were associated with dephosphorylation of cofilin and actin changes (33). Similarly, changes to the cell cytoskeleton are apparent after *C. neoformans* infection of *Drosophila* cells (34). Another example of close interaction, and possible manipulation of host cytoskeleton, is that in human endothelial cells transcytosis requires CD44 receptor, which triggers the movement of actin to membrane lipid rafts that presumably aid in cellular entry (35). Changes to the cytoskeleton are apparent before non-lytic exocytosis in the form of actin flashes (36).
6. *Non-lytic exocytosis and vacuolation*: non-lytic exocytosis is generally considered a relatively benign process on the host cell given that cells from which *C. neoformans* has exited can replicate (37, 38). However, it is likely that any interference in the process of non-lytic exocytosis will trigger lytic exocytosis (discussed below). In this regard, macrophage deficient in annexin A2, a protein that is involved in a myriad of cellular functions including membrane fusion and plasma membrane repair, manifested increased lytic exocytosis, possibly due to problems in membrane fusion during the process of exocytosis (39). Non-lytic exocytosis results in the formation of large vacuoles in the cytoplasm of macrophage from which *C. neoformans* exited (40). Although the mechanism and etiology of the formation of these large vacuoles is not known they represent an anatomic cellular abnormality that could interfere with cell homeostasis.
7. *Lytic exocytosis*: unchecked replication of *C. neoformans* inside macrophage can lead to rupture of the host cell with the release of fungal cells. This process, known as lytic exocytosis results in the death of the host cell. Host cell lysis by *C. neoformans* is also triggered by a mechanism that is dependent on cell wall mannosylation of cell wall and does not require the viability of the fungal cell (41).
8. *Activation of cell death pathways*: the toxic presence of *C. neoformans* may culminate in host cellular death. However, a study of death pathways activated in murine macrophage upon *C. neoformans* infection did not find a specific pathway predominantly activated. In contrast, in dendritic cells *C. neoformans* caused robust inflammasome activation and cell death, which was critically dependent of caspase-1, or caspase-8 in the absence of caspase-1 (28, 29). *C. neoformans* polysaccharides glucuronoxylomannan and galactoxylomannan can mediate direct cytoxicity to macrophage by activation of Fas ligand and triggering apoptosis (42, 43). Galatoxylomannan trigger B cell death leading to lymphocyte depletion and abrogating effective antibody responses (44).
9. *Extracellular vesicle (EV) effects. C. neoformans* like all other fungi that have been examined produces EVs (45). In the case of *C. neoformans*, these vesicles contain many fungal products associated with virulence, including fungal secreted polysaccharides and laccase (46). EVs are not directly toxic to host cells but they can have powerful stimulatory effects on host macrophage (47). In fact, incubation of macrophage with *C. neoformans* EVs led to activation changes that increased the antifungal activity (47). Although at this time EVs are not linked directly to cryptococcal pathogenesis there is considerable indirect evidence for an important role in virulence. For example, capsular polysaccharides have been implicated in negative effects on immune function, and this material is exported across the cryptococcal cell wall in EVs (5, 48). In addition to capsular polysaccharides, EVs have been shown to carry numerous short RNA molecules raising the possibility that these microbial RNAs are involved in modulating host cell function (49).

## Tissue and Organ Damage

### Immune System Damage

The immune system is damaged during *C. neoformans* infection by direct injury to its effector cells and by interference with effective immunity. The outcome of the *C. neoformans*–macrophage interaction is a critical determinant for the fate of the microbe and host during infection. The ability of *C. neoformans* to replicate inside macrophage correlated with mice and rat susceptibility to infection (50, 51). In humans, the capacity of *C. neoformans* strains to replicate in macrophage to higher intracellular burden correlated with worse clinical outcomes (52, 53). Hence, the available evidence suggests that factors and interventions that modulate macrophage function, in particular when T-cell function is impaired, could control cryptococcal disease, whereas the capacity of the yeast to efficiently replicate intracellularly is associated with progression of infection. In this light, it is apparent that mechanisms that damage macrophage are likely to impair the antifungal capacity of these cells, which in turn facilitates intracellular growth. Hence, mitochondrial damage, phagosomal damage, and induction of programmed cellular pathways can be expected to directly aid in fungal survival *in vivo* through impairment of monocyte mononuclear macrophage as well as other innate immune cells. Damage to other immune cells has been less studied, but for example direct effects of shed polysaccharides on adaptive cellular responses (54) will magnify the impairment to macrophage function by providing inadequate activation of microbicidal capacity. A last point is that shear physical force of capsule and cell body growth, to dimensions surpassing 10 μm, may exhaust intracellular membranes of the host and that this fungal gigantism could physically damage host cell (55, 56), as seen with capsule growth and titan cell formation.

The second form of damage to the immune system is interference with its ability to organize an effective response. Here, the damage is multifaceted and originates from the cellular damage described above as well direct effects of cryptococcal components that affect the response of immune cells, which in turn interfere with effective immunity. The major cryptococcal polysaccharides have protean effects on the function of immune cells, which contribute to dysregulated process [reviewed in Ref. (57)]. In addition, to polysaccharide-mediated effects, the presence of the *C. neoformans* urease in the lung promotes the accumulation of immature dendritic cells and the emergence of a non-protective T2 polarized inflammatory response (58). *C. neoformans* produces a variety of prostaglandins and leukotrienes, which have direct effects on inflammatory cells and thus may have a major effect in altering the local immune response to infection (3, 41). Synthesis of eicosanoids is dependent on phospholipase activity thus implicating this enzyme in several different possible mechanism of virulence (47). Cryptococcal polysaccharides interfere with leukocyte migration toward chemoattractants (59). The mechanism for this effect includes induction of L-selectin shedding from neutrophils (60). Interference with leukocyte migration could account for the notoriously poor inflammatory responses observed in many individuals (61).

The immune response to *C. neoformans* can also mediate host damage. This phenomenon was first when HIV-infected individuals successfully treated for cryptococcosis manifested a worsening of symptoms after the initiation of antiretroviral therapy (62, 63). What came to be known as “immune reconstitution inflammatory syndrome” was the result of immune system recovery reacting to residual cryptococcal antigens in tissue, which resulted in inflammation and local organ damage (63). Recently, T cells have been associated with immune injury in experimental murine cryptococcosis establishing a mechanism by which immune dysregulation in response to infection can produce host damage (3). The contribution of immune-mediated damage to the pathogenesis of cryptococcosis could help explain the paradoxical observation that the prognosis of cryptococcal meningitis if more favorable in patients with HIV infection and severe immunodeficiency than in those without obvious immune impairment (4). There is also some evidence that cryptococcal infection in the lung predisposes the host to develop allergic inflammation that could progress to hyperreactive airway diseases, such as asthma (64–66).

### Damage to BBB

The major cause of mortality and morbidity during cryptococcosis is meningoencephalitis. For *C. neoformans* to invade the central nervous system yeast cells must cross the BBB. *C. neoformans* crosses the BBB by two mechanisms: transcytosis, whereby yeast cells transit directly through endothelial cells and a Trojan Horse-like mechanism involving carriage inside an infected macrophage (67–69). The former mechanism involves undermining the integrity of the BBB (70) and is enhanced by brain inositol (71). The yeasts are trapped in the brain capillaries because of their size, allowing for active transcytosis (12). For the efficient Trojan horse crossing, *C. neoformans* must survive inside macrophage and, as noted above, fungal-mediated damage to the phagocytic cell enhances intracellularly cryptococcal survival. However, it is still poorly understood if the crossing of the BBB merely causes a transient disruption in integrity of the BBB or whether it has more pernicious consequences. One could hypothesize that entrapment of yeast in brain capillaries cause ischemia to surrounding tissues but this issue has not been formally addressed.

### Tissue Masses

A distinctive feature of many cases of cryptococcal meningoencephalitis is the formation of masses of yeast cells in the brain with little or no inflammation (Figure 1). This feature distinctively distinguishes these structures from granuloma where inflammation and immune response are well organized. These structures are so distinctive that they have been referred as “soap bubbles” as they are composed of gelatinous pseudocysts composed of packed *C. neoformans* cells with a particular appearance in magnetic resonance imaging (72). For masses of *C. neoformans* to form in the brain, they must grow in a manner that displaces or destroys brain tissue to create the space for the fungal mass. Given the propensity of *C. neoformans* to replicate inside cells and trigger host cell death such soap bubble anatomic lesions could be the result of progressive lysis of host cells at the fungal–brain interface. In this regard, *C. neoformans* can replicate inside microglial cells, the brain resident macrophage population (73). Alternatively, it is possible that such lesions represent fungal replication that creates spaces in the brain through compression of brain tissue through the force generated by fungal replication. Hence, irrespective of the mechanism of formation, soap bubble lesions represent *prima facie* evidence of direct fungal damage to brain tissue.

## Organism Damage

At the organism level the combination of molecular, cellular, and tissue damage leads to cryptococcosis (Figure 1). The damage–response framework of microbial pathogenesis posits that disease occurs when host damage is sufficient to affect hemostasis, which in turn produces clinical symptoms (74). For *C. neoformans* infections, host damage can come from both the microbe, as reviewed in this essay, and from the immune response (3, 4, 75). Although a discussion of how tissue damage results in clinical signs and symptoms that can ultimately lead to death is beyond the scope of this review, there is a clear connection between the types of damage discussed here and the disease.

## Relation of Cytotoxicity to Environmental Selection Pressures—Amoeba

Evolution of *C. neoformans* virulence, virulence being defined as capacity to survive or to cause disease in mammalian hosts, was proposed to arise from selection pressures in the environment by phagocytic predators such as amoeba (76, 77). According to this view, *C. neoformans* virulence factors needed for animal pathogenicity function emerged as characteristics that protect fungal cells against phagocytic predators. For example, the capsule, melanin, and phospholipase each contribute to fungal cell survival when preyed upon by amoeba (76). The outcome of amoeba–*C. neoformans* interactions is highly dependent on the conditions of the experiment. In conditions where there are minimal nutrients such as phosphate-buffered saline, *C. neoformans* is ascendant but the reverse occurs when there are nutrients for amoeba (48). The presence of extracellular Ca^2+^ and Mg^2+^ is enough to tilt the balance of the host–*C. neoformans* and allow amoeba to kill a significant portion of *C. neoformans* (78). Although far less is known about how *C. neoformans* damages amoeba than for mammalian cells, it is likely to have parallels in the mechanisms for cytotoxicity. In this regard, accumulation of polysaccharide-containing vesicles was observed in the cytoplasm of amoeba that ingested *C. neoformans* (76).

## A Synthesis for *C. neoformans*-Mediated Host Damage

For the purposes of this essay, we have considered host damage as a function of size scales but it is important to stress that damage is continuous from the molecular to organism level (Figure 1). Disseminated cryptococcosis is a rare disease in hosts with intact immunity, which means that host defense mechanisms are highly effective at confiding damage form inhaled *C. neoformans* to the molecular and cellular level in the lungs, such that damage does not rise to the level where homeostasis is affected and clinical symptoms ensue. Since cryptococcal infection is common and diseases is rare, and *C. neoformans* are common in the environment, it is likely that repeated cycles of macrophage infection occur in the lives of human hosts. Although we do not know the sequence of events that follow these interactions, the fact that these are asymptomatic suggests fungal control in the lung with minimal tissue damage. However, once there is impairment to the immune system, commonly following immunosuppression, HIV infection or iatrogenic, cryptococcal infection transforms from silent or latent, to a slow but inexorable progressive condition that invariably kills the host without aggressive therapy. However, more than a half century after the introduction of the first antifungal agent in the form of amphotericin B, the mortality and morbidity of cryptococcosis remains stubbornly high. Improvements in therapy may require a better understanding of the mechanisms of host damage that will allow the development of new therapeutic interventions. A critical synthesis of how the various types of host damage synergize to impair tissue function is an important next step for understanding the pathogenesis of cryptococcosis.

## Author Contributions

All authors listed have made a substantial, direct, and intellectual contribution to the work and approved it for publication.

## Conflict of Interest Statement

The authors declare that the research was conducted in the absence of any commercial or financial relationships that could be construed as a potential conflict of interest.

## References

[1] Luo Y Alvarez M Xia L Casadevall A. The outcome of phagocytic cell division with infectious cargo depends on single phagosome formation. PLoS One (2008) 3:e3219. 10.1371/journal.pone.0003219 18795151 PMC2535564

[2] Robertson EJ Najjuka G Rolfes MA Akampurira A Jain N Anantharanjit J *Cryptococcus neoformans* ex vivo capsule size is associated with intracranial pressure and host immune response in HIV-associated cryptococcal meningitis. J Infect Dis (2014) 209:74–82. 10.1093/infdis/jit435 23945372 PMC3864387

[3] Neal LM Xing E Xu J Kolbe JL Osterholzer JJ Segal BM CD4(+) T cells orchestrate lethal immune pathology despite fungal clearance during *Cryptococcus neoformans* meningoencephalitis. MBio (2017) 8:e01415–7. 10.1128/mBio.01415-17 29162707 PMC5698549

[4] Pirofski LA Casadevall A Immune-mediated damage completes the parabola: *Cryptococcus neoformans* pathogenesis can reflect the outcome of a weak or strong immune response. MBio (2017) 8:e02063-17. 10.1128/mBio.02063-17 PMC5727418 29233901

[5] Ganendren R Carter E Sorrell T Widmer F Wright L. Phospholipase B activity enhances adhesion of *Cryptococcus neoformans* to a human lung epithelial cell line. Microbes Infect (2006) 8:1006–15. 10.1016/j.micinf.2005.10.018 16487740

[6] Cox GM Mcdade HC Chen SC Tucker SC Gottfredsson M Wright LC Extracellular phospholipase activity is a virulence factor for *Cryptococcus neoformans*. Mol Microbiol (2001) 39:166–75. 10.1046/j.1365-2958.2001.02236.x 11123698

[7] Almeida F Wolf JM Casadevall A. Virulence-associated enzymes of *Cryptococ-cus neoformans*. Eukaryot Cell (2015) 14:1173–85. 10.1128/EC.00103-15 26453651 PMC4664877

[8] Chen L-C Blank E Casadevall A. Extracellular proteinase activity of *Cryptococcus neoformans*. Clin Diagn Lab Immunol (1996) 3:570–4. 8877137 10.1128/cdli.3.5.570-574.1996 PMC170408

[9] Xu CY Zhu HM Wu JH Wen H Liu CJ. Increased permeability of blood-brain barrier is mediated by serine protease during *Cryptococcus* meningitis. J Int Med Res (2014) 42:85–92. 10.1177/0300060513504365 24398759

[10] Homer CM Summers DK Goranov AI Clarke SC Wiesner DL Diedrich JK Intracellular action of a secreted peptide required for fungal virulence. Cell Host Microbe (2016) 19:849–64. 10.1016/j.chom.2016.05.001 27212659 PMC5186401

[11] Cox GM Mukherjee J Cole GT Casadevall A Perfect JR. Urease as a virulence factor in experimental cryptococcosis. Infect Immun (2000) 68:443–8. 10.1128/IAI.68.2.443-448.2000 10639402 PMC97161

[12] Shi M Li SS Zheng C Kim KS Zhou H Kubes P Real-time imaging of trapping and urease-dependent transmigration of *Cryptococcus* in the brain. J Clin Invest (2010) 120:1683–93. 10.1172/JCI41963 20424328 PMC2860939

[13] Chen SC Wright LC Santangelo RT Muller M Moran VR Kuchel PW Identification of extracellular phospholipase B, lysophospholipase, and acyltransferase produced by *Cryptococcus neoformans*. Infect Immun (1997) 65:405–11. 9009289 10.1128/iai.65.2.405-411.1997 PMC174609

[14] Santangelo R Zoellner H Sorrell T Wilson C Donald C Djordjevic J Role of extracellular phospholipases and mononuclear phagocytes in dissemination of cryptococcosis in a murine model. Infect Immun (2004) 72:2229–39. 10.1128/IAI.72.4.2229-2239.2004 15039347 PMC375158

[15] Shea JM Kechichian TB Luberto C Del Poeta M. The cryptococcal enzyme inositol phosphosphingolipid-phospholipase C confers resistance to the antifungal effects of macrophages and promotes fungal dissemination to the central nervous system. Infect Immun (2006) 74:5977–88. 10.1128/IAI.00768-06 16988277 PMC1594881

[16] Chayakulkeeree M Sorrell TC Siafakas AR Wilson CF Pantarat N Gerik KJ Role and mechanism of phosphatidylinositol-specific phospholipase C in survival and virulence of *Cryptococcus neoformans*. Mol Microbiol (2008) 69:809–26. 10.1111/j.1365-2958.2008.06310.x 18532984

[17] Djordjevic JT. Role of phospholipases in fungal fitness, pathogenicity, and drug development – lessons from *Cryptococcus neoformans*. Front Microbiol (2010) 1:125. 10.3389/fmicb.2010.00125 21687772 PMC3109512

[18] Pandey A Ding SL Qin QM Gupta R Gomez G Lin F Global reprogramming of host kinase signaling in response to fungal infection. Cell Host Microbe (2017) 21:637–49.e636. 10.1016/j.chom.2017.04.008 28494245 PMC5538893

[19] Taylor-Smith LM. *Cryptococcus*-epithelial interactions. J Fungi (Basel) (2017) 3:E53. 10.3390/jof3040053 29371569 PMC5753155

[20] Schelenz S Malhotra R Sim RB Holmskov U Bancroft GJ Binding of host collectins to the pathogenic yeast *Cryptococcus neoformans*: human surfactant D acts as an agglutinin for acapsular cells. Infect Immun (1995) 63:3360–6. 7642263 10.1128/iai.63.9.3360-3366.1995 PMC173462

[21] Drummond RA. Neuro-immune mechanisms of anti-cryptococcal protection. J Fungi (Basel) (2017) 4:E4. 10.3390/jof4010004 29371497 PMC5872307

[22] Levitz SM Nong SH Seetoo KF Harrison TS Speizer RA Simons ER. *Cryptococcus neoformans* resides in an acidic phagolysosome of human macrophages. Infect Immun (1999) 67:885–90. 9916104 10.1128/iai.67.2.885-890.1999 PMC96400

[23] Smith LM Dixon EF May RC. The fungal pathogen *Cryptococcus neoformans* manipulates macrophage phagosome maturation. Cell Microbiol (2015) 17:702–13. 10.1111/cmi.12394 25394938

[24] Feldmesser M Kress Y Novikoff P Casadevall A. *Cryptococcus neoformans* is a facultative intracellular pathogen in murine pulmonary infection. Infect Immun (2000) 68:4225–37. 10.1128/IAI.68.7.4225-4237.2000 10858240 PMC101732

[25] Tucker SC Casadevall A. Replication of *Cryptococcus neoformans* in macrophages is accompanied by phagosomal permeabilization and accumulation of vesicles containing polysaccharide in the cytoplasm. Proc Natl Acad Sci U S A (2002) 99:3165–70. 10.1073/pnas.052702799 11880650 PMC122490

[26] Chen M Xing Y Lu A Fang W Sun B Chen C Internalized *Cryptococcus neoformans* activates the canonical caspase-1 and the noncanonical caspase-8 inflammasomes. J Immunol (2015) 195:4962–72. 10.4049/jimmunol.1500865 26466953

[27] Davis MJ Eastman AJ Qiu Y Gregorka B Kozel TR Osterholzer JJ *Cryptococcus neoformans*-induced macrophage lysosome damage crucially contributes to fungal virulence. J Immunol (2015) 194:2219–31. 10.4049/jimmunol.1402376 25637026 PMC4379045

[28] Ben-Abdallah M Sturny-Leclere A Ave P Louise A Moyrand F Weih F Fungal-induced cell cycle impairment, chromosome instability and apoptosis via differential activation of NF-kappaB. PLoS Pathog (2012) 8:e1002555 10.1371/journal.ppat.1002555 22396644 PMC3291658

[29] Coelho C Souza AC Derengowski Lda S De Leon-Rodriguez C Wang B Leon-Rivera R Macrophage mitochondrial and stress response to ingestion of *Cryptococcus neoformans*. J Immunol (2015) 194:2345–57. 10.4049/jimmunol.1402350 25646306 PMC4340727

[30] Hirano A Zimmerman HM Levine S Fine structure of cerebral fluid accumulation. V. Transfer of fluid from extracellular compartments in acute phase of cryptococcal polysaccharide lesions. Arch Neurol (1964) 11:632–41. 10.1001/archneur.1964.00460240064009 14202180

[31] Hirano A Zimmerman HM Levine S The fine structure of cerebral fluid accumulation. III. Extracellular spread of cryptococcal polysaccharides in the acute stage. Am J Pathol (1964) 45:1–19. 14172716 PMC1907071

[32] Hirano A Zimmerman HM Levine S The fine structure of cerebral fluid accumulation. VII. Reactions of astrocytes to cryptococcal polysaccharide implantation. J Neuropathol Exp Neurol (1965) 24:386–96. 10.1097/00005072-196507000-00002

[33] Chen SH Stins MF Huang SH Chen YH Kwon-Chung KJ Chang Y *Cryptococcus neoformans* induces alterations in the cytoskeleton of human brain microvascular endothelial cells. J Med Microbiol (2003) 52:961–70. 10.1099/jmm.0.05230-0 14532340

[34] Qin QM Luo J Lin X Pei J Li L Ficht TA Functional analysis of host factors that mediate the intracellular lifestyle of *Cryptococcus neoformans*. PLoS Pathog (2011) 7:e1002078. 10.1371/journal.ppat.1002078 21698225 PMC3116820

[35] Jong A Wu CH Shackleford GM Kwon-Chung KJ Chang YC Chen HM Involvement of human CD44 during *Cryptococcus neoformans* infection of brain microvascular endothelial cells. Cell Microbiol (2008) 10:1313–26. 10.1111/j.1462-5822.2008.01128.x 18248627

[36] Johnston SA May RC. The human fungal pathogen *Cryptococcus neoformans* escapes macrophages by a phagosome emptying mechanism that is inhibited by Arp2/3 complex-mediated actin polymerisation. PLoS Pathog (2010) 6:e1001041. 10.1371/journal.ppat.1001041 20714349 PMC2920849

[37] Alvarez M Casadevall A Phagosome fusion and extrusion, and host cell survival following *Cryptococcus neoformans* phagocytosis by macrophages. Curr Biol (2006) 16:2161–5. 10.1016/j.cub.2006.09.061 17084702

[38] Ma H Croudace JE Lammas DA May RC. Expulsion of live pathogenic yeast by macrophages. Curr Biol (2006) 16:2156–60. 10.1016/j.cub.2006.09.032 17084701

[39] Stukes S Coelho C Rivera J Jedlicka AE Hajjar KA Casadevall A. The membrane phospholipid binding protein annexin A2 promotes phagocytosis and nonlytic exocytosis of *Cryptococcus neoformans* and impacts survival in fungal infection. J Immunol (2016) 197(4):1252–61. 10.4049/jimmunol.1501855 27371724 PMC5160961

[40] Alvarez M Casadevall A. Cell-to-cell spread and massive vacuole formation after *Cryptococcus neoformans* infection of murine macrophages. BMC Immunol (2007) 8:16. 10.1186/1471-2172-8-16 17705844 PMC1988836

[41] O’Meara TR Veri AO Ketela T Jiang B Roemer T Cowen LE. Global analysis of fungal morphology exposes mechanisms of host cell escape. Nat Commun (2015) 6:6741. 10.1038/ncomms7741 25824284 PMC4382923

[42] Pericolini E Cenci E Monari C De Jesus M Bistoni F Casadevall A *Cryptococcus neoformans* capsular polysaccharide component galactoxylomannan induces apoptosis of human T-cells through activation of caspase-8. Cell Microbiol (2006) 8:267–75. 10.1111/j.1462-5822.2005.00619.x 16441437

[43] Monari C Paganelli F Bistoni F Kozel TR Vecchiarelli A. Capsular polysaccharide induction of apoptosis by intrinsic and extrinsic mechanisms. Cell Microbiol (2008) 10:2129–37. 10.1111/j.1462-5822.2008.01196.x 18647312

[44] De JM Nicola AM Frases S Lee IR Mieses S Casadevall A. Galactoxylomannan-mediated immunological paralysis results from specific B cell depletion in the context of widespread immune system damage. J Immunol (2009) 183:3885–95. 10.4049/jimmunol.0900449 19684080 PMC2737596

[45] Rodrigues ML Nimrichter L Oliveira DL Frases S Miranda K Zaragoza O Vesicular polysaccharide export in *Cryptococcus neoformans* is a eukaryotic solution to the problem of fungal trans-cell wall transport. Eukaryot Cell (2007) 6:48–59. 10.1128/EC.00318-06 17114598 PMC1800364

[46] Rodrigues ML Nakayasu ES Oliveira DL Nimrichter L Nosanchuk JD Almeida IC Extracellular vesicles produced by *Cryptococcus neoformans* contain protein components associated with virulence. Eukaryot Cell (2008) 7:58–67. 10.1128/EC.00370-07 18039940 PMC2224146

[47] Oliveira DL Freire-De-Lima CG Nosanchuk JD Casadevall A Rodrigues ML Nimrichter L. Extracellular vesicles from *Cryptococcus neoformans* modulate macrophage functions. Infect Immun (2010) 78:1601–9. 10.1128/IAI.01171-09 20145096 PMC2849392

[48] Garcia-Solache MA Izquierdo-Garcia D Smith C Bergman A Casadevall A. Fungal virulence in a lepidopteran model is an emergent property with deterministic features. MBio (2013) 4:e00100–13. 10.1128/mBio.00100-13 23631914 PMC3648900

[49] Peres da Silva R Puccia R Rodrigues ML Oliveira DL Joffe LS Cesar GV Extracellular vesicle-mediated export of fungal RNA. Sci Rep (2015) 5:7763. 10.1038/srep07763 25586039 PMC5379013

[50] Shao X Mednick A Alvarez M Van Rooijen N Casadevall A Goldman DL. An innate immune system cell is a major determinant of species-related susceptibility differences to fungal pneumonia. J Immunol (2005) 175:3244–51. 10.4049/jimmunol.175.5.3244 16116215

[51] Zaragoza O Alvarez M Telzak A Rivera J Casadevall A. The relative susceptibility of mouse strains to pulmonary *Cryptococcus neoformans* infection is associated with pleiotropic differences in the immune response. Infect Immun (2007) 75:2729–39. 10.1128/IAI.00094-07 17371865 PMC1932903

[52] Alanio A Desnos-Ollivier M Dromer F. Dynamics of *Cryptococcus neoformans*-macrophage interactions reveal that fungal background influences outcome during cryptococcal meningoencephalitis in humans. MBio (2011) 2:e00158–111. 10.1128/mBio.00158-11 21828220 PMC3149853

[53] Sabiiti W Robertson E Beale MA Johnston SA Brouwer AE Loyse A Efficient phagocytosis and laccase activity affect the outcome of HIV-associated cryptococcosis. J Clin Invest (2014) 124:2000–8. 10.1172/JCI72950 24743149 PMC4001551

[54] Yauch LE Lam JS Levitz SM. Direct inhibition of T-cell responses by the *Cryptococcus* capsular polysaccharide glucuronoxylomannan. PLoS Pathog (2006) 2:e120. 10.1371/journal.ppat.0020120 17096589 PMC1635532

[55] Zaragoza O García-Rodas R Nosanchuk JD Cuenca-Estrella M Rodriguez-Tudela JL Casadevall A. Fungal cell gigantism during mammalian infection. PLoS Pathog (2010) 6:e1000945. 10.1371/journal.ppat.1000945 20585557 PMC2887474

[56] Hommel B Mukaremera L Cordero RJB Coelho C Desjardins CA Sturny-Leclère A Titan cells formation in *Cryptococcus neoformans* is finely tuned by environmental conditions and modulated by positive and negative genetic regulators. PLoS Pathog (2018) (in press). 10.1371/journal.ppat.1006982 PMC5959062 29775480

[57] Vecchiarelli A Pericolini E Gabrielli E Kenno S Perito S Cenci E Elucidating the immunological function of the *Cryptococcus neoformans* capsule. Future Microbiol (2013) 8:1107–16. 10.2217/fmb.13.84 24020739

[58] Osterholzer JJ Surana R Milam JE Montano GT Chen GH Sonstein J Cryptococcal urease promotes the accumulation of immature dendritic cells and a non-protective T2 immune response within the lung. Am J Pathol (2009) 174:932–43. 10.2353/ajpath.2009.080673 19218345 PMC2665753

[59] Ellerbroek PM Walenkamp AME Hoepelman AIM Coenjaerts FEJ Effects of the capsular polysaccharides of Cryptococcus neoformans on phagocyte migration and inflammatory mediators. Curr Med Chem (2004) 11(2):253–66. 14754421 10.2174/0929867043456188

[60] Dong ZM Murphy JW Cryptococcal polysaccharides induce L-selectin shedding and tumor necrosis factor receptor loss from the surface of human neutrophils. J Clin Invest (1996) 97:689–98. 10.1172/JCI118466 8609224 PMC507105

[61] Casadevall A Perfect JR Cryptococcus Neoformans. Washington, DC: ASM Press (1998).

[62] Lortholary O Fontanet A Memain N Martin A Sitbon K Dromer F Incidence and risk factors of immune reconstitution inflammatory syndrome complicating HIV-associated cryptococcosis in France. AIDS (2005) 19:1043–9. 10.1097/01.aids.0000174450.70874.30 15958835

[63] Beatty GW. Immune reconstitution inflammatory syndrome. Emerg Med Clin North Am (2010) 28:393–407. 10.1016/j.emc.2010.01.004 20413021

[64] Goldman DL Davis J Bommartio F Shao X Casadevall A Enhanced allergic inflammation and airway responsiveness in rats with chronic *Cryptococcus neoformans* infection suggests a potential role for fungal pulmonary infection in the pathogenesis of asthma. J Infect Dis (2006) 193:1178–86. 10.1086/501363 16544260

[65] Goldman DL Vicencio AG. The chitin connection. MBio (2012) 3:e00056–12. 10.1128/mBio.00056-12 22448043 PMC3315704

[66] Grahnert A Muller U Von Buttlar H Treudler R Alber G. Analysis of asthma patients for cryptococcal seroreactivity in an urban German area. Med Mycol (2015) 53:576–86. 10.1093/mmy/myv024 26026172

[67] Charlier C Nielsen K Daou S Brigitte M Chretien F Dromer F. Evidence of a role for monocytes in dissemination and brain invasion by *Cryptococcus neoformans*. Infect Immun (2009) 77:120–7. 10.1128/IAI.01065-08 18936186 PMC2612285

[68] Sorrell TC Juillard PG Djordjevic JT Kaufman-Francis K Dietmann A Milonig A Cryptococcal transmigration across a model brain blood-barrier: evidence of the Trojan horse mechanism and differences between *Cryptococcus neoformans* var. grubii strain H99 and *Cryptococcus gattii* strain R265. Microbes Infect (2016) 18:57–67. 10.1016/j.micinf.2015.08.017 26369713

[69] Santiago-Tirado FH Onken MD Cooper JA Klein RS Doering TL. Trojan horse transit contributes to blood-brain barrier crossing of a eukaryotic pathogen. MBio (2017) 8:e2183–2116. 10.1128/mBio.02183-16 28143979 PMC5285505

[70] Chang YC Stins MF Mccaffery MJ Miller GF Pare DR Dam T Cryptococ-cal yeast cells invade the central nervous system via transcellular penetration of the blood-brain barrier. Infect Immun (2004) 72:4985–95. 10.1128/IAI.72.11.6753.2004 15321990 PMC517459

[71] Liu TB Kim JC Wang Y Toffaletti DL Eugenin E Perfect JR Brain inositol is a novel stimulator for promoting *Cryptococcus* penetration of the blood-brain barrier. PLoS Pathog (2013) 9:e1003247. 10.1371/journal.ppat.1003247 23592982 PMC3617100

[72] Vieira MA Costa CH Ribeiro JC Nunes-Filho LP Rabelo MG Almeida-Neto WS. Soap bubble appearance in brain magnetic resonance imaging: cryptococcal meningoencephalitis. Rev Soc Bras Med Trop (2013) 46:658–9. 10.1590/0037-8682-0142-2013 24270259

[73] Lee SC Kress Y Zhao M-L Dickson DW Casadevall A *Cryptococcus neoformans* survive and replicate in spacious phagosomes in human microglia. Lab Invest (1995) 73:871–9. 8558850

[74] Casadevall A Pirofski L The damage-response framework of microbial pathogenesis. Nat Microbiol Rev (2003) 1:17–24. 10.1038/nrmicro732 PMC7097162 15040176

[75] Panackal AA Williamson KC Van De Beek D Boulware DR Williamson PR. Fighting the monster: applying the host damage framework to human central nervous system infections. MBio (2016) 7:e1906–15. 10.1128/mBio.01906-15 26814182 PMC4742705

[76] Steenbergen JN Shuman HA Casadevall A. *Cryptococcus neoformans* interactions with amoebae suggest an explanation for its virulence and intracellular pathogenic strategy in macrophages. Proc Natl Acad Sci U S A (2001) 18:15245–50. 10.1073/pnas.261418798 11742090 PMC65014

[77] Casadevall A. Amoeba provide insight into the origin of virulence in pathogenic fungi. Adv Exp Med Biol (2012) 710:1–10. 10.1007/978-1-4419-5638-5_1 22127880

[78] Fu MS Casadevall A Divalent metal cations potentiate the predatory capacity of amoeba for *Cryptococcus neoformans*. Appl Environ Microbiol (2017) 84(3):e01717 10.1128/AEM.01717-17 PMC5772251 29150507

